# Long-term catheter management in the community: a population-based analysis of user characteristics, service utilisation and costs in England

**DOI:** 10.1017/S1463423624000021

**Published:** 2024-03-07

**Authors:** Heather Gage, Peter Williams, Miriam Avery, Catherine Murphy, Mandy Fader

**Affiliations:** 1 Surrey Health Economics Centre, Department of Clinical and Experimental Medicine, School of Economics, University of Surrey, Guildford, England; 2 Department of Mathematics and Physicas, University of Surrey, Guildford, England; 3 Continence Technology and Skin Health Group, School of Health Sciences, University of Southampton, Southampton, England; 4 Bladder and Bowel Management Research Group, School of Health Sciences, University of Southampton, Southampton, England

**Keywords:** community costs, long-term catheter users, management

## Abstract

**Background:**

Long-term urinary catheters are problematic and burdensome for patients, carers and health services. Nursing practice to improve the management of long-term urinary catheters has been held back by a lack of evidence to support policy and practice. Little is known about who uses a catheter long term and the resources and costs needed for their management. Understanding these costs will help to target innovations to improve care. There have been no substantial innovations to urinary catheters or their management recently and no publications to characterise users and costs.

**Aim:**

To describe long-term catheter users and explore catheter-related service use and costs in England.

**Methods:**

Descriptive information on the characteristics of catheter users and their use of services was obtained from: General Practice records (*n* = 607), district nursing records (*n* = 303), questionnaires to patients (*n* = 333) and triangulated, 2009–2012. Annual service costs (British pounds 2011) were computed.

**Findings:**

Most catheter users (59.6%) were men, nearly three-quarters (71.2%) were over 70 years and 60.8% used a urethral catheter. Women tended to be younger than men and more likely to use a suprapubic catheter. The services used most frequently over 12 months were general practitioner (by 63.1%) and out of hours services (43.0%); 15.5% accessed Accident and Emergency services for urgent catheter-related care. Hospital use accounted for nearly half (48.9%) of total health service costs (mainly due to inpatient stays by 13.6% of participants); catheter supplies/medications were next most costly (25.7%). Half of all costs were accounted for by 14.2% of users. The median annual cost of services used was £6.38, IQR: £344–£1324; district nursing services added approximately a further £200 per annum.

**Conclusions:**

Finding better ways to reduce catheter problems (e.g. blockage, infection) that cause unplanned visits, urgent or hospital care should be a priority to improve quality of life for long-term catheter users and reduce health service expenditure.

## Key messages

- Most long-term catheter users in the community are men and over 70 years of age; urethral catheters are used by about 60%
- Women who have long-term catheters tend to be younger than men, and more likely to have a neurological reason for using the catheter, and use a suprapubic catheter
- A small proportion of users account for a very high proportion of total health service costs
- Finding ways to reduce unplanned service use is likely to reduce costs and should be a priority

## Introduction

Caring for people with long-term urinary catheters is an important part of the work of primary care and community-based staff. On average, the community prevalence of long-term catheter use in the United Kingdom (UK) is around 0.14%, rising with age to 0.73% in people over 70 years and 1.22% in those over 80 years (Gage *et al*., 2017). Long-term catheters are problematic and burdensome for patients and carers; costs are incurred by health services for the provision of the catheters and associated accessories and for managing complications such as blockages, leakage and infections (Getliffe and Newton, 2006; Feneley *et al.*, 2012; Cottenden *et al.*, 2013). With few innovations in catheter care in many years, improvements to services for catheter users have been called for (Wilde *et al.*, 2010; Wilde *et al.*, 2013). Little is known, however, about the characteristics of the people who rely daily on catheters and the resources involved in their management to help target innovations and inform policy and practice.

This paper reports a retrospective population-based cross-sectional study of the characteristics of long-term catheter users, reasons for catheterisation and catheter-related service use in the community. Data were collected from two large areas in the south (Southampton, Portsmouth, Hampshire, Dorset, Wiltshire, Isle of Wight) and west (Bath, Bristol, Swindon, Gloucestershire and Somerset) of England. Although collected more than a decade ago, these findings are derived from a unique dataset that describes the users and costs of long-term catheter management more comprehensively than prior or subsequent studies.

## Materials and methods

A large dataset was created from three sources. First, general practices (primary care providers of general medical services in the National Health Service (NHS) in England) were invited to participate in the study. Those agreeing were recruited on a rolling basis between June 2009 and October 2010 and reimbursed for helping with data collection. Each practice provided the research team with an anonymised printout of the records of all patients identified as having a catheter prescription during the previous 12 months. Unique patient study numbers were assigned and linked to names and contact details in a separate spreadsheet kept by the practice. Data were extracted from the practice records of each patient by the research team including: gender, date of birth, reason for catheter, type of catheter, months with catheter (if less than 12), medical conditions, catheter-related contacts with General Practitioner (GP), community professionals, out of hours services and hospital (Accident and Emergency (A&E), out and inpatient) services, catheter-related supplies and medications provided by prescription. Out of hours services included phone calls and home visits by nurses, GPs, emergency care physicians, ambulances and paramedics.

Second, to capture district nurse activity, the general practices in the study provided the local district nursing teams with the names and study IDs of each long-term catheter user identified by the database searches. The nurses were asked to refer to their own records and complete a questionnaire about each patient which they returned to the research team using only the unique study identifier. Data requested included: socio-economic and demographic features, home carer status, medical conditions and catheter-related information (including type, management plan and supplies). On the basis of their knowledge of each patient, nurses also completed the Barthel measure of independence in activities of daily living which covers ten different activities and produces an overall score between 0 (totally dependent) and 100 (totally independent) (Mahoney and Barthel, 1965). Finally, nurses were asked to refer to their records and list all contacts with each patient in the last six months, with reason (scheduled and unscheduled catheter changes, blockages, washouts, treating infections, etc).

Third, data were gathered from long-term catheter users themselves by means of a questionnaire mailed to them by their GPs. The questionnaire requested information on catheter care and socio-economic, demographic and health history. It was also used to capture quality of life data from respondents which has been reported separately (Cotterill *et al.*, 2016) and is not covered in this paper. Participants were asked to return their completed questionnaires to the study team in a freepost envelope that recorded the participant’s unique study ID for the purpose of linking data across sources.

Prior to any data collection, a favourable opinion was obtained from the Southmead (Bristol) Local Research Ethics Committee.

### Analysis

Data from the three sources were combined in a master Excel database using patient study IDs. Only patients having an indwelling catheter for three months or more were included and the data were cleaned. For example, inconsistencies in data were checked and corrected. For the service use and prescription variables, information from the general practice records were considered more reliable and used in preference to data collected from patients. The reason for long-term catheter use by each patient was categorised as either due to a neurological condition (including stroke, multiple sclerosis, Parkinson’s, spina bifida) or a non-neurological cause (e.g. prostate problem, cancer treatments, obstetric accident) by reference to the general practice data.

The characteristics of the long-term catheter users were summarised descriptively. The gender, age, reason for catheter (neuro/ non neuro) and type of catheter (urethral or suprapubic) were available from general practice records and for the largest sample of patients. The district nurse records and patient questionnaires provided additional information on socio-economic characteristics, health and catheter management but was only available for subsets of the general practice sample due to non-response. Associations between key characteristics were explored using appropriate statistical tests (depending on type of variable).

The 12-month use of catheter-related health services extracted from general practice records were summarised descriptively and analysed. The cost of supplies, medications, contacts with services, tests and hospital care were calculated for each participant by applying nationally validated unit costs for 2011 (NHS Digital, 2010; Curtis, 2011) and totalled over a 12-month period (or annualised for participants with a catheter for less than 12 months). Costs were compared by sex, age group, reason for catheter (neuro/non neuro) and type of catheter (urethral/suprapubic) using unpaired t tests.

Associations between total costs and indicators of functioning abilities (living situation (alone vs with others), problems with self-care, self-management of catheter and having a visiting carer, Barthel score) were examined for the subset of participants with data from the patient or District Nurse (DN) questionnaires using Mann–Whitney U tests (Spearman’s rho for Barthel).

Catheter care activities from district nurse records were summarised descriptively over the six-month reporting period. Associations between the total number of catheter-related activities (planned and unscheduled) reported by nurses and selected patient characteristics (age, Barthel score) were examined using t tests and Pearson’s correlation.

## Results

### Response rates

Of a total of 724 practices (396 in the south and 328 in the west Primary Care Research Network areas), 51 practices volunteered to take part in the study (23 in the south, 28 in the west, providing data for 703 long-term catheter users. Data from five practices were excluded, one in the south (because of concerns that the practice staff had selectively not provided the research team with records of some long-term catheter users such as those with terminal illness) and four in the west (because changes in practice configurations caused uncertainties around the age and sex data extracted from the practice database).

**Figure 1.**
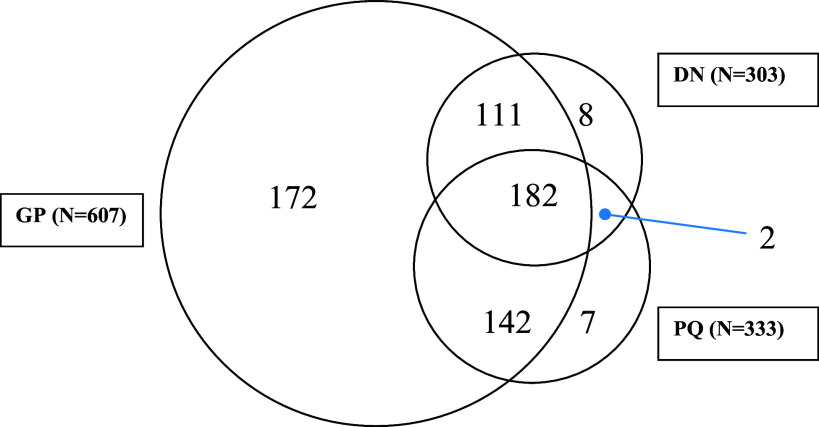
Venn diagram showing the number of participants with data from each of the three data sources, or combination of sources: general practice records (GP), District Nurse (DN) and Patient Questionnaire (PQ).

Patients returned 414 questionnaires and district nurses supplied data for 337 patients. After data cleaning to remove inconsistencies between sources, the final numbers of participants analysed from each source were general practice records, *n* = 607; patient questionnaires, *n* = 333; district nurse questionnaires, *n* = 303. The maximum number of participants available for analysis (i.e. had data from either general practice records or district nurse records or the patient questionnaire alone or in combination) was 624 (referred to as ALL). The number of respondents and overlaps between the sources are shown in Fig. 1.

### Characteristics of long-term catheter users

Most participants were male (59.6%), over 70 years of age (71.2%), had a catheter as a result of a neurological condition (57.0%) and were using a urethral (rather than suprapubic) catheter (60.8%). Most participants reported receiving welfare benefits, had some problems with self-care and lived with others. Almost one-half reported self-managing their catheter on a daily basis (Table 1). Compared to people who lived with others, those living alone were more likely to self-manage their catheters (63.0 % vs. 40.9%, Chi sq *P* <  0.0005) and to have a visiting carer (74.4% vs. 46.0%, Chi sq *P* < 0.0005).

**Table 1.** Characteristics of long-term catheter users

Characteristic	Data source	N	n	%
Sex: % male	ALL	607	362	59.6
Age < 70 years*	ALL	612	176	28.8
Reason for catheter: neuro (vs non neuro)	ALL	609	347	57.0
Type of catheter: urethral (vs suprapubic)	ALL	566	334	60.8
Education: % formal educated after 18 years	PQ	283	81	28.6
Received benefits (excludes child benefit, pension)	PQ	296	199	67.2
Time with catheter (1: <1; 2:1–5; 3:6–10; 4: >10 years)	PQ or DN	345	Median 1 to 5 yrs	
Lives alone (vs. with others)	PQ or DN	442	122	27.6
No problems with self-care (vs some or unable)	PQ	333	99	29.7
Self manages catheter – Yes	PQ or DN	446	210	47.1
Has visiting carer – Yes	PQ or DN	437	225	53.8
Wheelchair user – Yes	PQ or DN	446	256	57.4
Barthel level of independence^∼^	DN	303	Median 40	

* Mean 75.8, SD 14.7, median 79.5, range 19 –101; mean (SD) male 78.8(13.3) vs. female 71.4(15.5), *t* test *P* < 0.0005.

∼ Barthel range 0 dependent – 100 independent (Mahoney and Barthel, 1965).

**Figure 2.**
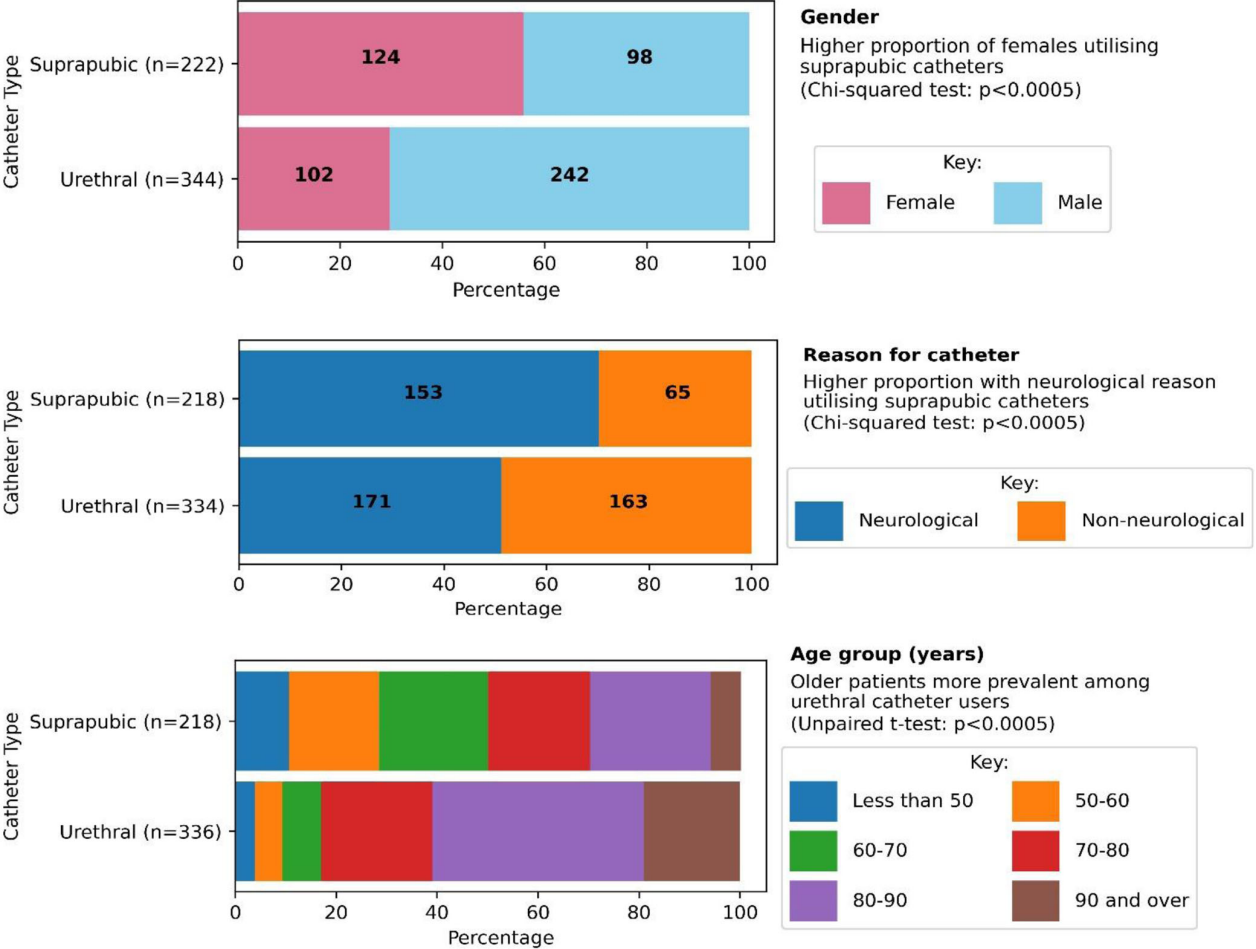
Breakdown of catheter type by gender, reason and age.

Comparing men and women, significantly higher proportions of women are in younger age groups, have catheters for neurological reasons and use a suprapubic catheter (Fig. 2, Supplementary Table 1).

### Twelve-month use of catheter-related health services and costs

**Figure 3.**
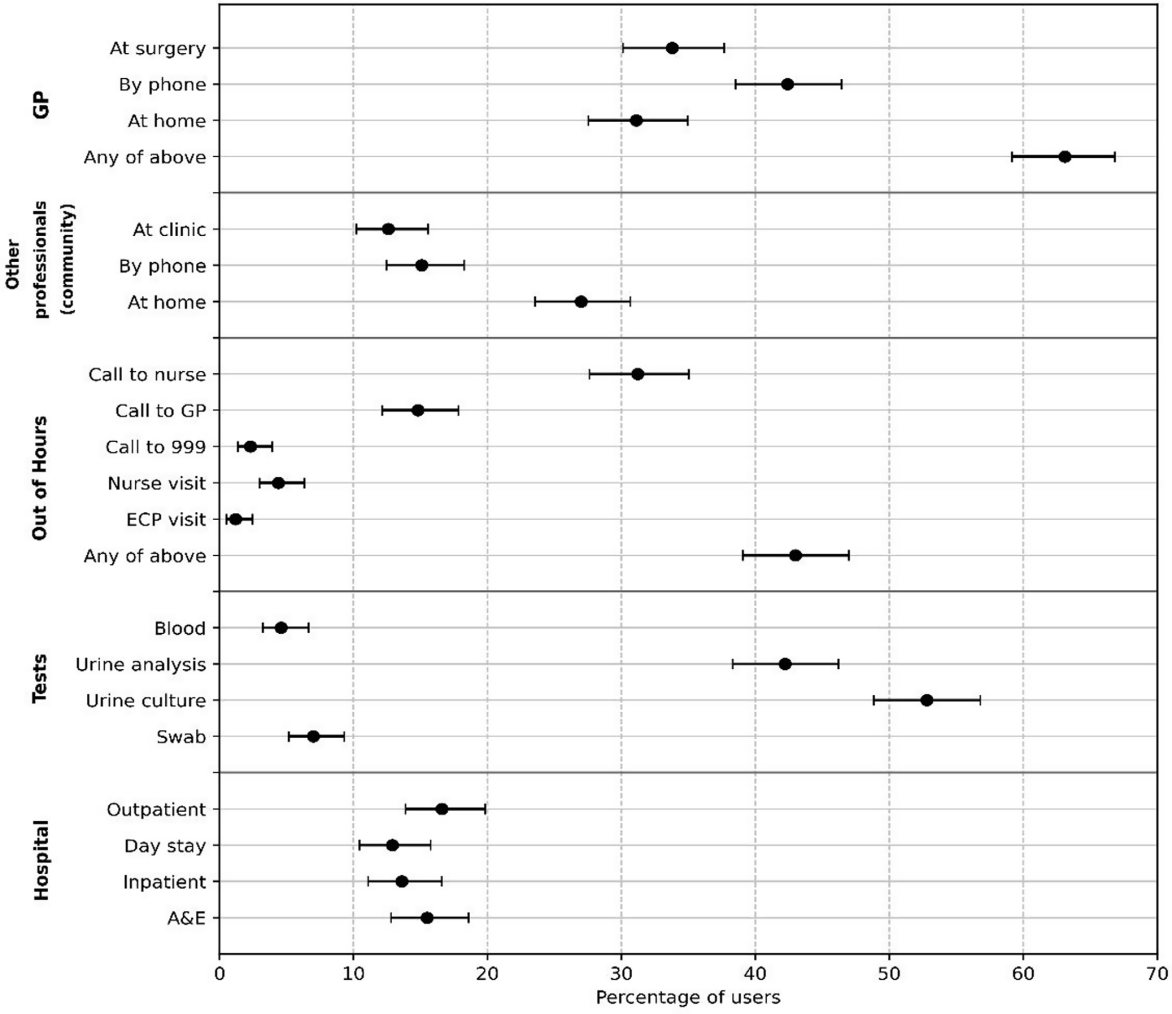
Service use by category: observed percentages with 95% confidence intervals. GP = General practitioner; ECP = Emergency Care Physician; 999 calls are to emergency services - ambulances and paramedics; A&E = Accident and Emergency department.

**Figure 4.**
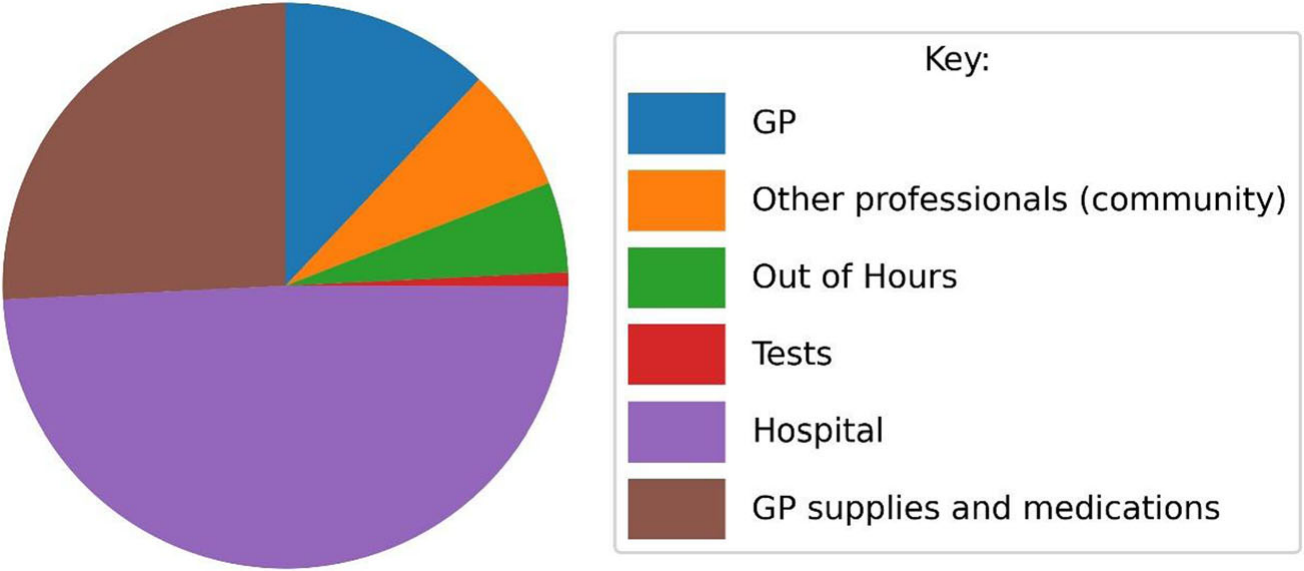
Proportion of total cost by category of service use.

**Figure 5.**
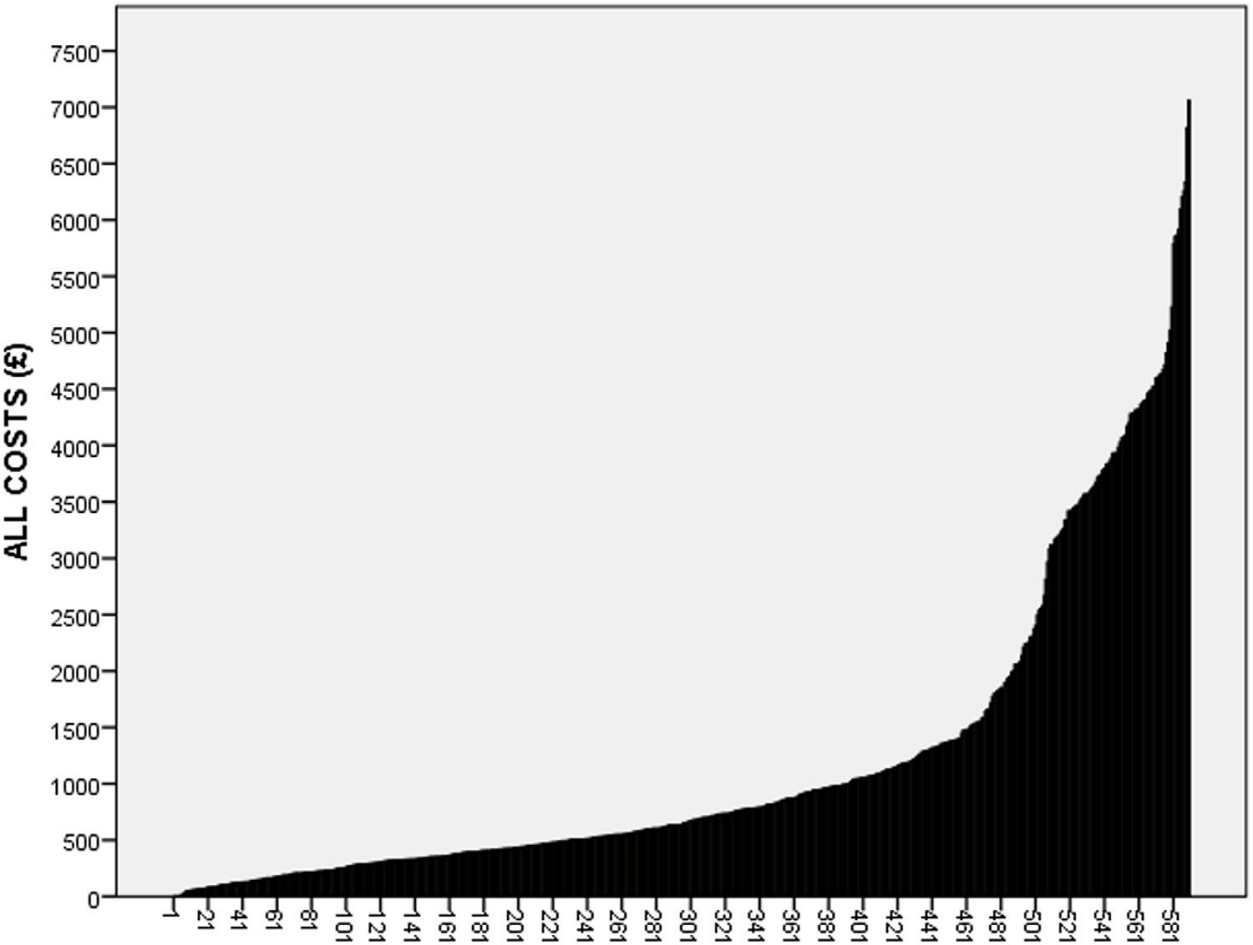
Total costs for 624 individual participants plotted in increasing order.

Based on the data from ALL records, the services used most frequently were GP, used by 63.1% of all participants, and out of hours services used by 43.0%. Inpatient episodes were recorded for 13.6% of the sample, but they accounted for nearly half (48.9%) of total cost (Figs. 3 and 4). Admissions were for a variety of complications, including infections, sepsis and problems arising from catheter blockages or suprapubic catheter insertion. A&E was accessed by 15.5% over the 12-month period. Supplies and medications (catheter related) prescribed in general practice were the next most costly item after hospital use and accounted for, on average, 25.7% of total costs. High expenditure on supplies was recorded for a small number of participants who had frequent washouts and replacement valves, drainage and leg bags. The mean annual total cost of services used was £1190.74, median £638 and Interquartile range (IQR) £344–£1324, but there was considerable variability between participants in all categories of costs (Fig. 5, Supplementary Table 2 and Supplementary Figure 1). The data showed a skewed distribution; 14.2% of catheter users account for 50% of costs. The most expensive 10% of users each cost 10 times more than a user in the least expensive 10% with regard to direct catheter costs and 20 times more if all costs (including hospital stays) are included.

Further examination of costs shows that men and older people have significantly higher costs than women for both GP and hospital utilisation, but significantly higher medication and supplies costs were incurred by women, younger participants, those with suprapubic catheters and those with neurological conditions (Supplementary Table 3).

### Six-month catheter-related care, from district nurse records and patient self-report

District nurse records showed that the average number of contacts with patients for catheter care was seven visits per annum, about two-thirds of which were planned, adding on average, about £200 per year to the costs of catheter management. Nurse reporting of catheter-related events suggested that long-term catheter users experienced, on average, 1.7 blockages, 0.35 leaks or dislodgements and 0.1 infections per annum. Patients responding to the patient questionnaire, however, reported that these events were more frequent. The highest users of district nurse services for catheter-related activity (planned plus unplanned) were younger patients (Pearson r for age: -.164, *P* = 0.004); those with higher levels of dependency (Barthel) (Pearson r: -.172, *P* = 0.003); those having a neurological condition (mean with vs. without neurological condition 3.78 vs 3.29, unpaired t test *P* = 0.002) and those using a suprapubic (rather than urethral) catheter (3.86 vs 3.27, unpaired t test *P* = 0.018).

## Discussion

This study fills a gap by providing descriptive information on the characteristics of community dwelling long-term catheter users and their use of services. Data from general practice records across two regions of England showed that 60% of people living in the community with a catheter for more than three months were men; 71% were over the age of 70. Suprapubic catheters were used by 39%. Women who had a long-term catheter tend to be younger than men and be more likely to have a neurological cause and to use a suprapubic catheter.

Long-term catheter users were found to draw frequently on primary and community services, including out of hours services, with many contacts being unplanned and arising from problems related to the catheter; a small proportions of users had several hospitalisations. Hospital use accounted for the largest proportion of total costs, but arose mostly from inpatient episodes by 13.6% of the sample. Provision of catheter supplies and medications was the next most costly item accounting for about one-fifth of total annual costs. There was high variability in costs at an individual level; older age, having a neurological condition and using a suprapubic catheter were associated with higher costs. A small group of users accounted for a high percentage of the costs. Based on a population prevalence of around 0.144, it is estimated that there are over 90 000 long-term catheter users in the UK (Gage *et al.*, 2017). Taking the average annual cost to the NHS of catheter management of £1191, with a further £200 for district nursing activity per patient per year, implies an annual expenditure of £125 million (2011).

### Strengths and limitations

Strengths of the study are that it provides population-based data from general practice records and that it enables an estimate of the costs of catheter management. Prior evidence on the characteristics of long-term catheter users and their service use tends to be based on selected samples. Since the NHS in England requires all members of society to register with a local GP, the use of general practice records to identify catheter users in this study was a population-based approach. Additionally, supplementing data from general practice records with information from district nurse records and the patients themselves sought to ensure that care from all providers of primary and community care was included, resulting in the accumulation of a large and unique dataset. Consistent with the results of a hospital-based audit in England (Tay *et al.*, 2016), the analysis found that over 15% of long-term catheter users attended A&E over a 12-month period. The findings also confirm the results of a pilot study in England which found large variability between individuals (Evans *et al.*, 2000).

The study has some limitations. The data were collected a number of years ago and relates to resource use at the time of collection. There have been no major changes to catheter designs or in catheter management practices, however, since then, although the NHS Cost Inflation index has risen year on year by about 2% (Jones and Burns, 2021). Not all general practices in the study areas volunteered to take part, and some patient records had to be excluded due to uncertainties in the data. The findings are based largely on the content of general practice records and therefore rely on those being complete and on the data extraction algorithms providing full information. People using catheters for more than three months but less than 12 months (i.e. between three and nine months) had service use and costs annualised, and this may have introduced some inaccuracies. District nurse data were only available for a sub-sample of patients. Inconsistencies between the three sources (general practice, district nurse and patient reported) occurred and when these could not be resolved from original sources, general practice data were taken as the default. There were disagreements between district nurse records and patient self-report in the questionnaires regarding the incidence of problems, such as blockages and infections, with patients typically reporting higher frequency than nurses. We do not know why this occurred. It could be that nurses did not record all events, or recall errors by patients. Moreover, a lack of granularity meant that data on blockages, leaks and infections were sometimes missing. The reason for the catheter was defined as either of neurological origin on non-neurological, whereas these categories are not necessarily mutually exclusive. The analysis relies on bivariate association methods, rather than multivariate because of associations between predictor variables (age, gender, reason for catheter and type of catheter).

### Implications for policy and practice

Catheter-related problems cause distress for patients, reduce quality of life and create additional expenditure for the health service. Exploring ways to reduce adverse effects with a focus on individuals with a high occurrence of unplanned service use is a priority. Unplanned catheter-related events occur regularly with 43% of participants in our study accessing out of hours services and 15% accessing A&E over the 12-month period. Moreover, one-third of district nurse visits were outside of routine scheduled care. Catheter care diaries have been suggested as a means to ensure catheter changes occur before a blockage arises (Getliffe, 2003), but practice differs internationally. For example, it is reported that routine catheter changes are more frequent in United States (US) (every four weeks) than United Kingdom (UK) (up to 12 weeks), and that more evidence is required on infection risks to inform protocols (Hooton *et al.*, 2010; McGoldrick, 2016). A self-management intervention in a US sample of long-term catheter users was not found to have any effect on blockages, infections and dislodgements (Wilde *et al.*, 2015). Evidence is lacking on how catheter-type impacts on complications and costs. Accordingly, a study in the UK has recently been launched comparing the traditional Foley catheters with Optitip catheters for infection frequency and cost-effectiveness. Data for this randomised controlled trial (CaDeT, https://www.southampton.ac.uk/ctu/trialportfolio/listoftrials/cadet.page) are being gathered prospectively by nurses in the field including catheter use, catheter complications and catheter-related service use. Participants will be followed for 12 months so that the health benefits and costs to health services of alternative regimens can be evaluated and guidance provided to practitioners.

## Supporting information

Gage et al. supplementary material Gage et al. supplementary material

## Supplementary material

To view supplementary material for this article, please visit https://doi.org/10.1017/S1463423624000021
