# Supplementary material for: Long-term catheter management in the community: a population-based analysis of user characteristics, service utilisation and costs in England
Source: Prim Health Care Res Dev. 2024 Mar 7;25:e13. doi: 10.1017/S1463423624000021 (PMC10940055; doi:10.1017/S1463423624000021)

**Supplementary Table 1: Associations between age, sex, reason for catheter use and type of catheter**

|  |  | **Catheter type** | | | | **Reason for catheter** | | | | **Age group in years, subsequently analysed as a continuous variable** | | | | | | | |
| --- | --- | --- | --- | --- | --- | --- | --- | --- | --- | --- | --- | --- | --- | --- | --- | --- | --- |
|  |  | **N** | **Urethral** | **Supra**  **pubic** | **Chi squared test**  **p value** | **N** | **Not neuro** | **Neurol**  **-ogical** | **Chi squared test**  **p value** | **N** | **Less than 50** | **50-59.99** | **60-69.99** | **70-79.99** | **80-89.99** | **90 and over** | **Unpaired t test**  **p value** |
| **Sex** | **Female** | 226 | 102  45.1% | 124  54.9% | <0.0005 | 245 | 71  29.0% | 174  71.0% | <0.0005 | 245 | 20  8.2% | 42  17.1% | 49  20.0% | 46  18.8% | 59  24.1% | 29  11.8% | <0.0005 |
|  | **Male** | 340 | 242  71.2% | 98  28.8% |  | 364 | 191  52.5% | 173  47.5% |  | 367 | 19  5.2% | 17  4.6% | 29  7.9% | 85  23.2% | 160  43.6% | 57  15.5% |  |
|  | **Total** | 566 | 344  60.8% | 222  39.2% |  | 609 | 262  43.0% | 347  57.0% |  | 612 | 39  6.4% | 59  9.6% | 78  12.7% | 131  21.4% | 219  35.8% | 86  14.1% |  |
|  |  |  |  |  |  |  |  |  |  |  |  |  |  |  |  |  |  |
| **Catheter type** | **Urethral** |  |  |  |  | 334 | 163  48.8 | 171  51.2% | <0.0005 | 336 | 13  3.9% | 18  5.4% | 26  7.7% | 74  22.0% | 141  42.0% | 64  19.0% | <0.0005 |
|  | **Supra**  **pubic** |  |  |  |  | 218 | 65  29.8% | 153  70.2% |  | 218 | 23  10.6% | 39  17.9% | 47  21.6% | 44  20.2% | 52  23.9% | 13  6.0% |  |
|  | **Total** |  |  |  |  | 552 | 228  41.3% | 324  58.7% |  | 554 | 36  6.5% | 57  10.3% | 73  13.2% | 118  21.3% | 193  34.8% | 77  13.9% |  |
|  |  |  |  |  |  |  |  |  |  |  |  |  |  |  |  |  |  |
| **Reason for catheter** | **Not neuro** |  |  |  |  |  |  |  |  | 257 | 5  1.9% | 3  1.2% | 19  7.4% | 61  23.7% | 118  45.9% | 51  19.8% | <0.0005 |
|  | **Neruol**  **-ogical** |  |  |  |  |  |  |  |  | 342 | 33  9.6% | 54  15.8% | 59  17.3% | 69  20.2% | 94  27.5% | 33  9.6% |  |
|  | **Total** |  |  |  |  |  |  |  |  | 599 | 38  6.3% | 57  9.5% | 78  13.0% | 130  21.7% | 212  35.4% | 84  14.0% |  |
|  |  |  |  |  |  |  |  |  |  |  |  |  |  |  |  |  |  |

**Supplementary Table 2: Catheter-related service use, and costs, over 12 months, N=624 people with long-term catheters, from GP records [249, 39.9% female; 375, 60.1% male]**

|  |  | **N** | **Zeros** | **Service Use** | | | | **Costs £2011** | | | |
| --- | --- | --- | --- | --- | --- | --- | --- | --- | --- | --- | --- |
| **Service** | **Type** |  | **N, %** | **Mean** | **Median** | **SD** | **IQR** | **Mean** | **Median** | **SD** | **IQR** |
| GP | At surgery | 601 | 398, 66.2% | 0.86 | 0 | 2.07 | 0 to 1 | 31.14 | 0 | 74.64 | 0 to 36 |
|  | By phone | 601 | 346, 57.6% | 1.34 | 0 | 2.68 | 0 to 2 | 29.57 | 0 | 59.01 | 0 to 44 |
|  | At home | 601 | 414, 68.9% | 0.67 | 0 | 1.51 | 0 to 1 | 81.56 | 0 | 182.22 | 0 to 121 |
|  | Any of above | 601 | 222, 36.9% |  |  |  |  |  |  |  |  |
| Total |  | 601 |  | 2.88 | 1 | 4.51 | 0 to 4 | 142.26 | 44 | 235.98 | 0 to 184.25 |
|  |  |  |  |  |  |  |  |  |  |  |  |
| Other professionals (community) | At clinic | 601 | 525, 87.4% | 0.76 | 0 | 5.63 | 0 to 0 | 29.52 | 0 | 21.62 | 0 to 0 |
|  | By phone | 601 | 510, 84.9% | 0.43 | 0 | 1.49 | 0 to 0 | 2.38 | 0 | 8.19 | 0 to 0 |
|  | At home | 601 | 439, 73.0% | 1.81 | 0 | 5.72 | 0 to 1 | 51.77 | 0 | 162.86 | 0 to 28.5 |
| Total |  | 601 |  |  |  |  |  | 83.67 | 0 | 275.18 | 0 to 56.94 |
|  |  |  |  |  |  |  |  |  |  |  |  |
| Out of Hours | Call to nurse | 596 | 410, 68.8% | 1.37 | 0 | 3.74 | 0 to 1 | 45.24 | 0 | 123.48 | 0 to 33 |
|  | Call to GP | 596 | 508, 85.2% | 0.25 | 0 | 0.72 | 0 to 0 | 8.13 | 0 | 23.88 | 0 to 0 |
|  | Call to 999 | 596 | 582, 97.7% | 0.03 | 0 | 0.23 | 0 to 0 | 0.97 | 0 | 7.02 | 0 to 0 |
|  | Nurse visit | 596 | 570, 95.6% | 0.06 | 0 | 0.34 | 0 to 0 | 4.54 | 0 | 24.47 | 0 to 0 |
|  | ECP visit | 596 | 589, 98.8% | 0.02 | 0 | 0.22 | 0 to 0 | 2.36 | 0 | 25.65 | 0 to 0 |
|  | Any of above | 596 | 340, 57.0% |  |  |  |  |  |  |  |  |
| Total |  | 596 |  | 1.73 | 0 | 3.93 | 0 to 2 | 61.27 | 0 | 133.77 | 0 to 66 |
|  |  |  |  |  |  |  |  |  |  |  |  |
| Tests | Blood | 603 | 575, 95.4% | 0.11 | 0 | 0.75 | 0 to 0 | 0.19 | 0 | 1.28 | 0 to 0 |
|  | Urine analysis | 602 | 348, 57.8% | 1.07 | 0 | 2.08 | 0 to 1 | 0.09 | 0 | 0.17 | 0 to 0.08 |
|  | Urine culture | 602 | 284, 47.2% | 1.61 | 0 | 2.58 | 0 to 2 | 7.59 | 4.71 | 12.16 | 0 to 9.42 |
|  | Swab | 602 | 560, 93.0% | 0.14 | 0 | 0.71 | 0 to 0 | 1.46 | 0 | 7.57 | 0 to 0 |
| Total |  | 602 |  |  |  |  |  | 9.32 | 4.71 | 15.7 | 0 to 11.50 |
|  |  |  |  |  |  |  |  |  |  |  |  |
| Hospital | Outpatient | 613 | 511*, 83.4% | 0.44 | 0 | 1.27 | 0 to 0 | 77.02 | 0 | 221.83 | 0 to 0 |
|  | Day stays | 613 | 534, 88.6% | 0.18 | 0 | 0.58 | 0 to 0 | 122.81 | 0 | 401.32 | 0 to 0 |
|  | Inpatient days | 602 | 520, 86.4% | 1.84 | 0 | 9.01 | 0 to 0 | 363.63 | 0 | 957.84 | 0 to 0 |
|  | A&E | 608 | 514, 84.5% | 0.22 | 0 | 0.60 | 0 to 0 | 23.94 | 0 | 63.94 | 0 to 0 |
| Total |  | 600 |  |  |  |  |  | 582.84 | 0 | 1181.84 | 0 to 511.50 |
|  |  |  |  |  |  |  |  |  |  |  |  |
| GP supplies and medications |  | 604 |  |  |  |  |  | 305.43 | 249.63 | 304.25 | 123.68 to 386.80 |
|  |  |  |  |  |  |  |  |  |  |  |  |
| ALL COSTS |  | 590 |  |  |  |  |  | 1190.74 | 637.73 | 1361.86 | 343.74 to 1324.04 |
| SD: Standard Deviation; IQR: Interquartile Range; * Never or less than once a year (because annual outpatient appointments are routine)  ^1^ Unit costs used in the calculations: GP consultations at practice (11.7 minutes) £36; GP phone calls (7.1 minutes) £22; GP home visit (23.4 minutes, includes travel) £121; GP nurse at practice (15.5 minutes) £39; other community professional (home visit 23.4 minutes, phone call 7 minutes) £28.50, £5.50 [Curtis, 2011]. Out of hours doctor home visit, nurse phone calls, nurse home visits based on previous unit costs with 1.5 multiplier for unsocial hours. All hospital services from NHS Reference costs 2011 [NHS Reference Costs, 2012]. Tests: blood £1.71 / test; urine analysis £0.08 / strip, culture £4.71; wound swab £10.60, from local hospital finance department. Medications and supplies from Prescription Cost Analysis, 2010 [NHS Digital, 2011]. | | | | | | | | | | | |

**Supplementary Table 3: Catheter related costs^1^, over 12 months, N=624 people with long term catheters, [249, 39.9% female; 375, 60.1% male]**

|  | Costs: | GP | | | Other Community | | | Out of Hours | | | Tests | | | Hospital | | | Medicines, Supplies | | | Total | | |
| --- | --- | --- | --- | --- | --- | --- | --- | --- | --- | --- | --- | --- | --- | --- | --- | --- | --- | --- | --- | --- | --- | --- |
|  |  | N | Mean  SD | 95% Cis | N | Mean  SD | 95% Cis | 95%   CIs | Mean  SD | 95% CIs | N | Mean  SD | 95% CIs | N | Mean  SD | 95%   CIs | N | Mean  SD | 95% CIs | N | Mean  SD | 95%   CIs |
| Sex | Female | 242 | 110.41 | 86.3 | 242 | 81.85 | 46.39 | 241 | 57.60 | 43.07 | 243 | 8.18 | 6.69 | 243 | 345.24 | 230.7 | 244 | 362.77 | 320.57 | 238 | 952.84 | 817.61 |
|  |  |  | 190.36 | 134.51 |  | 280.03 | 117.31 |  | 114.56 | 72.14 |  | 11.77 | 9.67 |  | 906.46 | 459.79 |  | 334.61 | 404.96 |  | 1059.01 | 1088.08 |
|  | Male | 359 | 163.74 | 136.71 | 359 | 84.90 | 56.64 | 355 | 63.72 | 48.53 | 359 | 10.10 | 8.25 | 357 | 744.57 | 607.79 | 360 | 266.56 | 238,00 | 352 | 1351.58 | 1192.93 |
|  |  |  | 260.35 | 190.76 |  | 272.25 | 113.15 |  | 145.47 | 78.9 |  | 17.85 | 11.95 |  | 1314.07 | 881.35 |  | 275.58 | 295.13 |  | 1513.49 | 1510.24 |
|  | Unpaired t test, p value | 0.004 | | | 0.894 | | | 0.584 | | | 0.112 | | | <0.0001 | | | <0.0001 | | | <0.0001 | | |
|  |  |  |  |  |  |  |  |  |  |  |  |  |  |  |  |  |  |  |  |  |  |  |
| Catheter type | Urethral | 328 | 156.17 | 131.70 | 328 | 91.80 | 60.32 | 325 | 71.52 | 55.44 | 330 | 9.18 | 7.69 | 328 | 564.89 | 434.23 | 330 | 268.46 | 239.57 | 323 | 1165.73 | 1015.00 |
|  |  |  | 225.29 | 180.64 |  | 289.84 | 123.29 |  | 147.39 | 87.61 |  | 13.70 | 10.66 |  | 1202.89 | 695.55 |  | 266.77 | 297.35 |  | 1376.95 | 1316.46 |
|  | Supra public | 215 | 122.67 | 87.97 | 215 | 86.76 | 48.18 | 213 | 52.38 | 35.83 | 214 | 10.20 | 7.63 | 214 | 617.11 | 460.54 | 216 | 370.50 | 323.24 | 209 | 1272.05 | 1087.16 |
|  |  |  | 258.19 | 157.38 |  | 287.04 | 125.35 |  | 122.54 | 68.93 |  | 19.10 | 12.77 |  | 1162.01 | 773.69 |  | 352.37 | 417.75 |  | 1355.80 | 1456.94 |
|  | Unpaired t test, p value | 0.111 | | | 0.842 | | | 0.116 | | | 0.467 | | | 0.617 | | | <0.0001 | | | 0.382 | | |
|  |  |  |  |  |  |  |  |  |  |  |  |  |  |  |  |  |  |  |  |  |  |  |
| Reason for catheter | Not neurological | 249 | 143.7 | 112.81 | 249 | 84.37 | 54.76 | 244 | 60.81 | 43.29 | 249 | 8.93 | 7.21 | 247 | 619.58 | 475.05 | 249 | 241.63 | 216.33 | 243 | 1163.05 | 994.11 |
|  |  |  | 247.54 | 174.60 |  | 237.20 | 113.97 |  | 138.99 | 78.35 |  | 13.79 | 10.65 |  | 1153.27 | 764.12 |  | 202.72 | 266.94 |  | 1336.93 | 1331.99 |
|  | Neurological | 345 | 137.76 | 114.18 | 345 | 84.67 | 52.66 | 345 | 62.12 | 48.20 | 346 | 9.45 | 7.68 | 346 | 559.56 | 431.37 | 348 | 351.60 | 314.22 | 340 | 1212.62 | 1064.06 |
|  |  |  | 222.68 | 161.34 |  | 302.32 | 116.69 |  | 131.38 | 76.03 |  | 16.76 | 11.23 |  | 1212.35 | 687.75 |  | 354.51 | 388.98 |  | 1392.71 | 1361.19 |
|  | Unpaired t test, p value | 0.76 | | | 0.989 | | | 0.908 | | | 0.688 | | | 0.544 | | | <0.0001 | | | 0.667 | | |
|  |  |  |  |  |  |  |  |  |  |  |  |  |  |  |  |  |  |  |  |  |  |  |
| Age in Years | Under 70 | 171 | 108.03 | 80.16 | 171 | 82.51 | 39.12 | 172 | 60.24 | 41.77 | 172 | 9.35 | 6.81 | 173 | 433.99 | 281.69 | 173 | 429.12 | 364.29 | 169 | 1123.71 | 940.35 |
|  |  |  | 184.67 | 135.91 |  | 287.42 | 125.89 |  | 122.74 | 78.71 |  | 16.88 | 11.89 |  | 1014.89 | 586.30 |  | 431.97 | 493.94 |  | 1207.43 | 1307.07 |
|  | ≥70 | 426 | 155.89 | 131.76 | 426 | 84.92 | 59.06 | 420 | 62.08 | 48.78 | 426 | 9.22 | 7.77 | 423 | 648.16 | 529.29 | 427 | 258.11 | 237.63 | 417 | 1226.35 | 1089.34 |
|  |  |  | 253.32 | 180.01 |  | 271.65 | 110.78 |  | 138.70 | 75.38 |  | 15.18 | 10.66 |  | 1243.76 | 767.03 |  | 215.38 | 276.61 |  | 1423.29 | 1363.35 |
|  | Unpaired t test, p value | 0.011 | | | 0.923 | | | 0.88 | | | 0.926 | | | 0.03 | | | <0.0001 | | | 0.377 | | |
|  | Spearman Rho* | Rho=0.051, p=0.215 | | | Rho=-0.009, p=0.826 | | | Rho=0.068, p=0.100 | | | Rho=0.10, p=0.812 | | | Rho=0.049, p=0.236 | | | Rho=-0.181, p<0.0001 | | | Rho=-0.026, p=0.532 | | |

* Age as continuous variable

^1^ Unit costs used in the calculations: GP consultations at practice (11.7 minutes) £36; GP phone calls (7.1 minutes) £22; GP home visit (23.4 minutes, includes travel) £121; GP nurse at practice (15.5 minutes) £39; other community professional (home visit 23.4 minutes, phone call 7 minutes) £28.50, £5.50 [Curtis, 2011]. Out of hours doctor home visit, nurse phone calls, nurse home visits based on previous unit costs with 1.5 multiplier for unsocial hours. All hospital services from NHS Reference costs 2011 [NHS Reference Costs, 2011]. Tests: blood £1.71 / test; urine analysis £0.08 / strip, culture £4.71; wound swab £10.60, from local hospital finance department. Medications and supplies from Prescription Cost Analysis, 2010 [NHS Digital, 2011].

**Supplementary Figure 1: Costs for 624 individual participants plotted in increasing order**


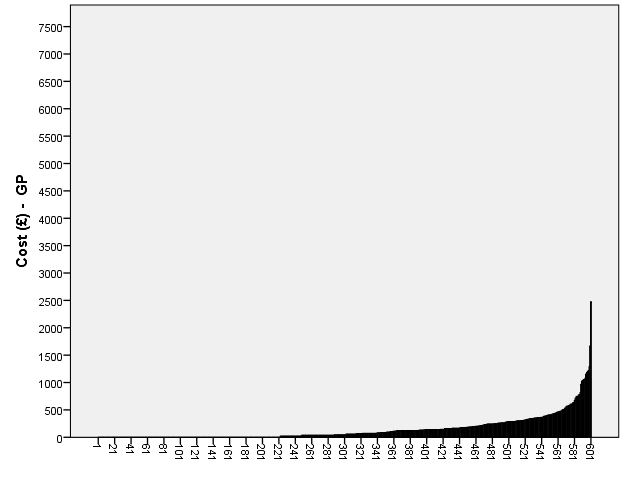

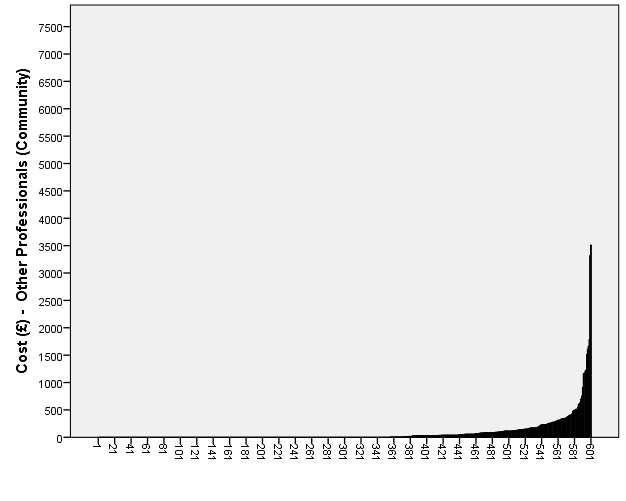

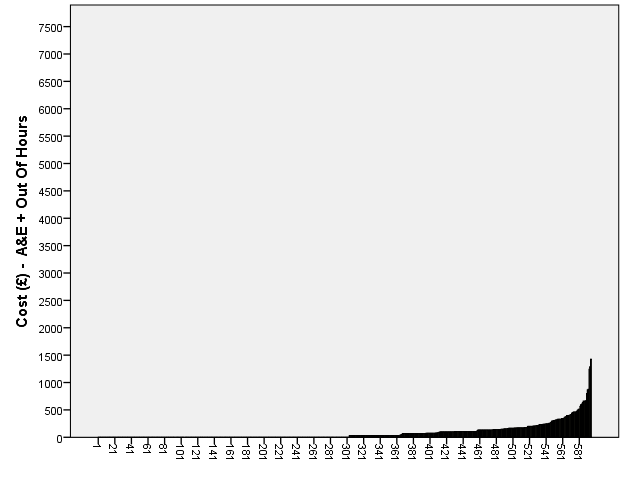

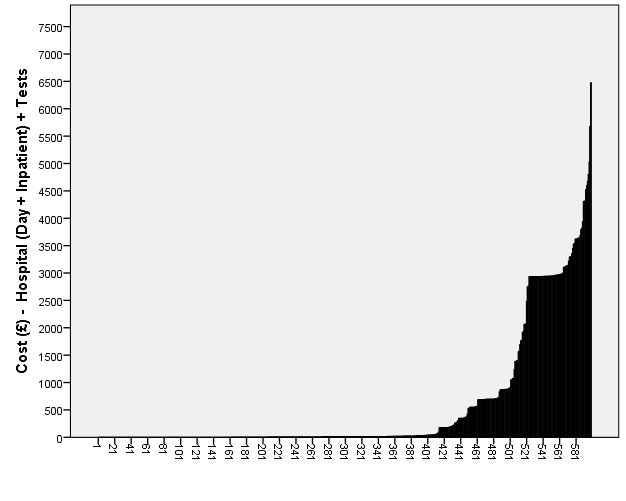

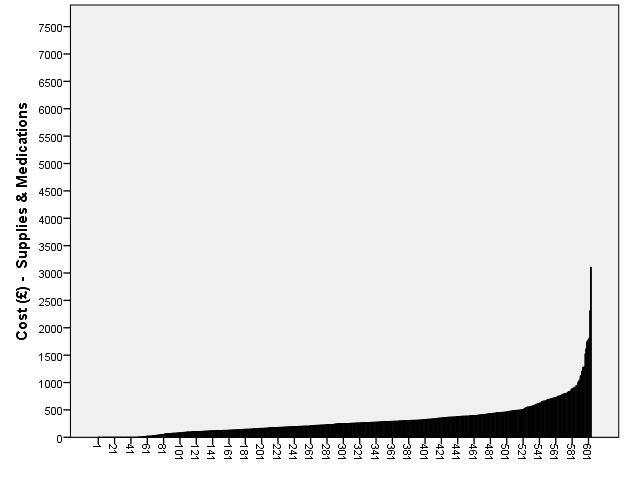

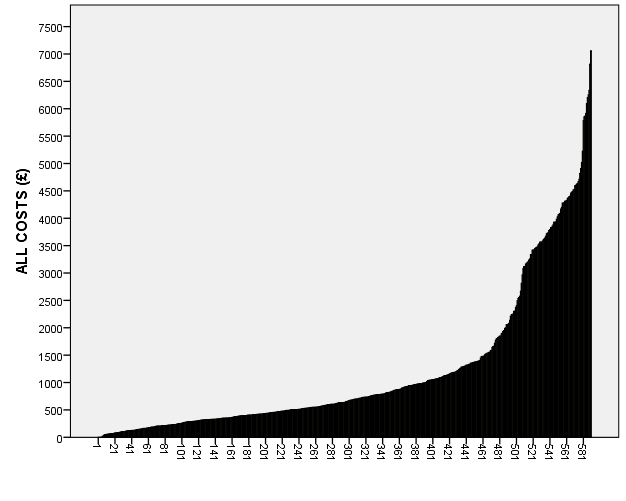

Supplement: Gage et al. supplementary material [file S1463423624000021sup001.docx]
